# Anemia as a risk factor for disease progression in patients admitted for COVID-19: data from a large, multicenter cohort study

**DOI:** 10.1038/s41598-023-36208-y

**Published:** 2023-06-03

**Authors:** Nicola Veronese, Francesco Vladimiro Segala, Luca Carruba, Anna La Carrubba, Francesco Pollicino, Giusi Di Franco, Giacomo Guido, Mariangela Cormio, Alessia Lugli, Laura De Santis, Vittorio Guerra, Martino Pepe, Rocco Tritto, Marco Matteo Ciccone, Davide Fiore Bavaro, Gaetano Brindicci, Pasquale Mansueto, Lydia Giannitrapani, Francesco Di Gennaro, Mario Barbagallo, Annalisa Saracino

**Affiliations:** 1 Geriatric Unit, Department of Internal Medicine and Geriatrics, University of Palermo, Via del Vespro 141, 90127 Palermo, Italy; 2 Clinic of Infectious Diseases, Department of Precision and Regenerative Medicine and Ionian Area (DiMePRe-J), University of Bari Aldo Moro, 70124 Bari, Italy; 3 Section of Cardiovascular Diseases, Department of Interdisciplinary Medicine (DIM), University of Bari Aldo Moro, Bari, Italy; 4 Institute for Biomedical Research and Innovation (IRIB), National Research Council (CNR), Via Ugo La Malfa 153, 90146 Palermo, Italy

**Keywords:** Health care, Medical research, Pathogenesis, Risk factors, Haematological diseases, Respiratory tract diseases, Diseases, Infectious diseases, Viral infection

## Abstract

In respiratory infections, anemia is both a consequence of acute inflammation and a predictor of poor clinical outcomes. There are few studies investigating the role of anemia in COVID-19, suggesting a potential role in predicting disease severity. In this study, we aimed to assess the association between the presence of anemia at admission and incidence of severe disease and death in patients hospitalized for COVID-19. Data from all adult patients admitted for COVID-19 in University Hospital “P. Giaccone” Palermo, and University Hospital of Bari, Italy, were retrospectively collected from 1st of September 2020 to 31 August 2022. The association between anemia (defined as Hb < 13 g/dl and < 12 g/dl in males and females, respectively), in-hospital mortality and severe COVID-19 was tested using a Cox’s regression analysis. Severe COVID-19 forms were defined as admission to intensive or sub-intensive care unit or a qSOFAscore ≥ 2 or CURB65scores ≥ 3. *p* values were calculated using the Student’s *t* test for continuous variables and the Mantel–Haenszel Chi-square test for categorical ones. The association between anemia and the mortality was made using a Cox’s regression analysis, adjusted, in two models, for the potential confounders and using a propensity score. Among the 1562 patients included in the analysis, prevalence of anemia was 45.1% (95% CI 43–48%). Patients with anemia were significantly older (*p* < 0.0001), reported more co-morbidities, and presented higher baseline levels of procalcitonin, CRP, ferritin and IL-6. Overall, the crude incidence of mortality was about four times higher in patients with anemia compared to those without. After adjusting for 17 potential confounders, the presence of anemia significantly increased the risk of death (HR = 2.68; 95% CI: 1.59–4.52) and of risk of severe COVID-19 (OR = 2.31; 95% CI: 1.65–3.24). The propensity score analysis substantially confirmed these analyses. Our study provides evidence that, in patients hospitalized for COVID-19, anemia is both associated with a more pronounced baseline pro-inflammatory profile and higher incidence of in-hospital mortality and severe disease.

## Introduction

The advent of the COVID-19 pandemic, a highly transmissible zoonotic infection caused by SARS-CoV2, has resulted in a global health crisis not witnessed since the 1918 Spanish Flu Influenza pandemic^1^ and consequences for both patients and healthcare workers^2^. Since clinical manifestations include a wide spectrum of signs and symptoms^3^—ranging from asymptomatic infections to acute respiratory distress syndrome and death—considerable efforts have been devoted to identifying the subset of patients at highest risk of disease progression^4,5^. Hence, in some patients, COVID-19 infection triggers a broad dysregulation of the host response to infection that encompasses overproduction of pro-inflammatory cytokines, disruption of the coagulation pathway, and abnormal endothelial activation^6^.

In COVID-19 patients, the overexpression of interleukin (IL)-1, IL-6, interferon-γ and tumor necrosis factor (TNF)-α profoundly affect iron metabolism and erythropoiesis, leading to shortened erythrocyte lifespan, segregation of circulating iron into macrophages in the bone marrow, liver and spleen and shifting blood cell production towards myelopoiesis at the expense of erythropoiesis^7^ and finally leading to anemia. In addition to the effects of inflammation, the drop in hemoglobin levels may be due also to a thrombotic microangiopathy caused by direct, viral-mediated endothelial injury (endothelitis), which in turn causes ADAMTS-13 consumption, activation of the intrinsic coagulation pathway and microvascular hemolytic anemia^8,9^. Both pathways, inflammation and intravascular coagulopathy, are supported by the characteristic laboratory abnormalities typically seen in severe COVID-19 patients, such as hyperferritinemia, leukocytopenia, thrombocytopenia, C-reactive protein, IL-6^10^ and D-dimer level^11^.

As seen in other lower respiratory tract infections, anaemia is both common and independently associated with poor clinical outcomes^12^. However, even as we enter the third year since the advent of the pandemic, evidence about prevalence and prognostic role of anemia in patients hospitalised for COVID-19 is still conflicting, controlled for a restricted number of covariates, and mainly based on monocentric studies with low numerosity^13–15^. In this study, we aimed to assess the association between the presence of anemia at admission and incidence of severe disease and death in patients hospitalized for COVID-19 in a large cohort of patients in two Teaching Hospitals in Southern Italy.

## Materials and methods

### Study population

All patients aging > 18 years and hospitalized in Internal Medicine or Geriatrics Wards from 01st September 2020 in the University Hospital (Policlinico) ‘P. Giaccone’ in Palermo, Sicily, Italy^16^ and in the University Hospital Policlinico (Bari, Italy) were enrolled in this study. The study conducted in Palermo was approved by the Local Ethical Committee during the session of the 28th of April 2021 (Comitato Etico Policlinico “P. Giaccone”, number 04/2021) and in Bari 28 April 2020 (Comitato Etico Policlinico di Bari, Study Code: 6357). Written informed consent was obtained from participants. The study is in accordance with Declaration of Helsinki/ relevant institutional guidelines.

### Exposure: anemia

Hemoglobin (Hb) levels were measured according to standard tools in Palermo and Bari and reported in g/dl, during the first day of hospitalization. Anemia was defined according to the World Health Organization (WHO) definition as Hb concentration of less than 13 g/dl and 12 g/dl in males and females, respectively^17^.

### Outcomes: mortality and severe COVID-19

Mortality was recorded during hospitalization using death certificates and medical records. Severe COVID-19 forms were defined as admission to intensive care unit or a sub-intensive care unit, such as pneumology, or a qSOFAscore ≥ 2 or CURB65scores ≥ 3. Briefly, the qSOFA (quick SOFA) is composed by three items, i.e., altered mental status, respiratory rate > 22, systolic blood pressure less than 100 mmHg. A score over two indicates a higher risk due to sepsis^18^. The CURB-65 (Confusion, Urea, Respiratory rate, Blood pressure, and age over 65 years) is used for estimating mortality associated with pneumonia. A score over 3 overall indicates severe forms of pneumonia^19^.

### Clinical and bio-humoral confounders

For better understanding the association between anemia and the outcomes of interest, we included several potential confounders, such as:

- Demographic characteristics, including age, gender, smoking status;
- Comorbidities, including hypertension, dyslipidemia, diabetes mellitus, and renal failure. These medical conditions were diagnosed using medical history, drug history, laboratory measures recorded in the first four days of hospitalization.
- Signs and symptoms typical of COVID-19 such as fever, anosmia etc. recorded at hospital admission;
- Laboratory measures, recorded in the first four days of hospital admission according to standard measurements;
- Respiratory parameters, such as the presence of pneumonia at the CT or x-ray scan, use of Venturi’s mask or high flow oxygen during hospitalization.

### Statistical analysis

We analyzed the normality of the continuous variables using the Kolmogorov–Smirnov test. Continuous variables are represented by means and standard deviation (SD) values. Percentages were used for the categorical variables, according to the presence or not of anemia at hospital admission. The homoscedasticity of variances was analyzed using the Levene’s test; Welch’s ANOVA was used if its assumption was not satisfied. *p* values were calculated using the Student’s T-test for independent samples for continuous variables and the Chi-square test for categorical ones.

The association between anemia and the mortality was made using a Cox’s regression analysis, adjusted, in two models, for the potential confounders and using a propensity score. All the factors included in the multivariable model were significantly different between anemia and non-anemia group or associated with mortality in univariate analyses, as *p*-values < 0.05. The collinearity among factors was analyzed using the variance inflaction factor^19^ over two, but no factor was excluded for this reason. To minimize the effect of potential confounders, we used a propensity score matching with one case (anemia) and one control (no anemia). The results were reported as hazard ratios (HRs) with their 95% confidence intervals (95% CI). The association between anemia and severe forms of COVID-19 as defined before, was analyzed using a logistic regression analysis (since no date to event was available) and the data reported as odds ratios (ORs) with 95% CI.

All analyses were performed using the SPSS 26.0 for Windows (SPSS Inc., Chicago, Illinois) and STATA 14.0. All statistical tests were two-tailed and statistical significance was assumed for a *p*-value < 0.05.

## Results

Among 1,666 patients initially included, we excluded 47 since no data regarding Hb levels and 57 since no data regarding outcomes of interest were available. Overall, the prevalence of anemia was 45.1% (95% CI between 43 and 48%).

As shown in Table 1, the 705 patients with anemia were significantly older (*p* < 0.0001), whilst no differences in terms of females’ presence was present (*p* = 0.74) compared to the 857 patients without anemia. Patients with anemia reported a significantly higher presence of comorbidities (particularly hypertension, diabetes, renal failure) than their counterparts. However, patients with anemia reported a significant lower prevalence of COVID-19 signs and symptoms (such as dyspnea, anosmia, fever, and gastrointestinal symptoms such as vomit or diarrhea) than their counterparts. Patients with anemia had a significantly higher prevalence of low oxygen saturation and a pro-inflammatory profile than patients without anemia and a higher prevalence of high levels of troponin and D-dimer. Finally, during the hospitalization, patients with anemia reported a higher risk of use of Venturi’s mask or high-flow oxygen than patients without anemia (Table 1).

**Table 1:** Descriptive characteristics at the baseline, stratified by presence or not of anemia.

Parameter (n = 1562)	No anemia (n = 857)	Anemia (n = 705)	*p*-value
Age, mean (± SD)	57.1 (16.0)	60.2 (16.4)	< 0.0001
Females, n (%)	372 (43.4)	**312** (44.3)	0.74
Comorbidities, n (%)			
At least one comorbidity	339 (39.6)	416 (59.0)	< 0.0001
Hypertension	360 (42.0)	351 (49.8)	0.002
Actual smoking	86 (10.0)	68 (9.7)	0.313
Previous smoking	32 (3.7)	17 (2.4)	
Dyslipidemia	99 (11.6)	80 (11.3)	0.9
Diabetes mellitus	161 (18.8)	176 (24.9)	< 0.0001
Renal failure	32 (3.7)	223 (10.1)	< 0.0001
Clinical presentation, n (%)			
Dyspnea	373 (43.5)	21 (31.6)	< 0.0001
Anosmia	59 (6.9)	71 (3.0)	< 0.0001
Dysgeusia	117 (13.7)	177 (25.1)	< 0.0001
Fever	609 (71.1)	379 (53.8)	< 0.0001
Cough	318 (37.1)	253 (35.9)	0.610
Gastrointestinal symptoms	179 (20.9)	238 (33.8)	< 0.0001
Oxygen saturation < 92% (%)	150 (17.5)	247 (35.0)	< 0.0001
Laboratory parameters, n (%)			
Elevated Procalcitonin	148 (17.3)	253 (35.9)	< 0.0001
Elevated D-Dimer	501 (58.5)	446 (63.3)	0.011
Elevated CRP	688 (80.3)	633 (89.8)	< 0.0001
Elevated Troponin	97 (11.3)	120 (17.0)	< 0.0001
Elevated Transaminases	132 (15.4)	107 (15.2)	0.99
Elevated IL6	372 (43.4)	293 (41.6)	< 0.0001
Elevated Ferritine	294 (34.3)	331 (47.0)	< 0.0001
Elevated LDH	252 (29.4)	252 (35.7)	< 0.0001
Low platelets levels	122 (14.2)	96 (13.6)	0.725
Presence of pneumonia at the CT scan or chest X-ray, n (%)	803 (93.7)	675 (95.7)	0.074
Use of Venturi’s mask during hospitalization, n (%)	440 (51.3)	568 (80.6)	< 0.0001
Use of high flow oxygen, n (%)	156 (18.2)	456 (64.7)	< 0.0001

*CRP* C-reactive protein; *CT* computed tomography; *IL-6* interleukin-6; *LDH* lactate de-hydrogenase

Significant values are in [bold].

**Figure 1:**
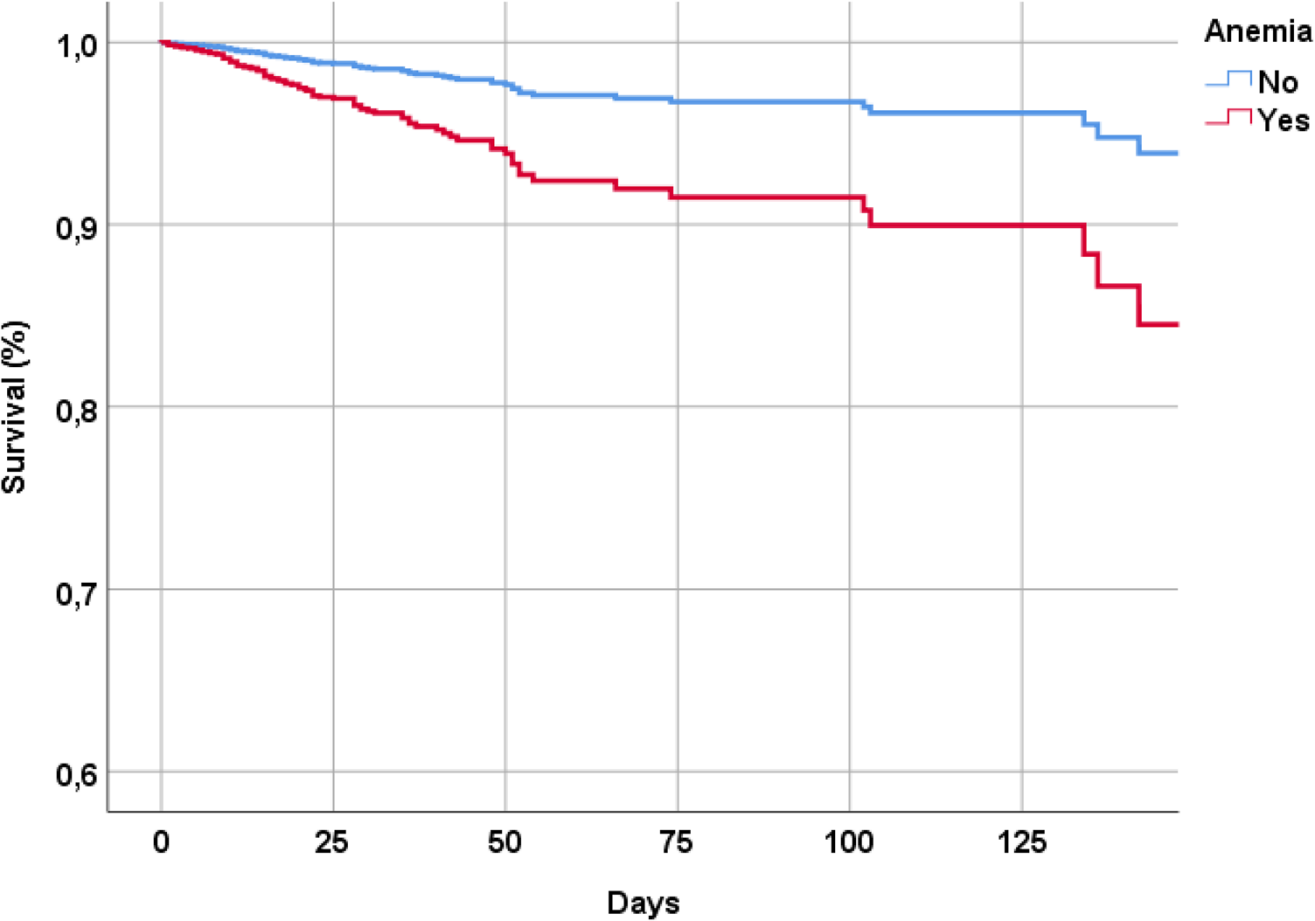
Survival curve by presence of anemia at the hospital admission.

During a median of 15 days of hospitalization, 113 patients (= 7.3%) died and 931 (= 59.6%) reported a severe form of COVID-19. As shown in Fig. 1, patients with anemia reported a higher incidence of mortality compared to patients without this condition. Table 2 reports the incidence of the outcomes of interest in people with and without anemia, in the sample as whole and after using a propensity score matching. Overall, the crude incidence of mortality was about four times higher in patients with anemia compared to those without. After adjusting for 17 potential confounders, the presence of anemia significantly increased the risk of death (HR = 2.68; 95% CI: 1.59–4.52) and of risk of severe COVID-19 (OR = 2.31; 95% CI: 1.65–3.24) (Table 2). The propensity score analysis substantially confirmed these analyses.

**Table 2:** Association between anemia at hospital admission and outcomes of interest.

	No anemia	Anemia	
		All sample	Propensity score^4^
Mortality, incidence rate^1^	243 (135–439)	934 (610–1434)	–
Risk of mortality, model 1^2^	1, reference	3.19 (1.97–5.14)	–
Risk of mortality, model 2^3^	1, reference	2.68 (1.59–4.52)	1.91 (1.10–3.32)
Risk of severe COVID-19, model 1^2^	1, reference	6.59 (5.11–8.49)	–
Risk of severe COVID-19, model 2^3^	1, reference	2.31 (1.65–3.24)	1.77 (1.26–2.48)

^1^Data are reported as incidence rates (with 95% confidence intervals) per 100,000 persons-year.

^2^The data are reported as hazard ratio for mortality and as odds ratio (with 95% confidence intervals) for severe COVID-19, adjusted for age, sex, center.

^3^The data are reported as hazard ratio for mortality and as odds ratio (with 95% confidence intervals) for severe COVID-19, adjusted for variables in Model 1 and presence of comorbidity, smoking status, oxygen saturation < 92%, elevated procalcitonin, elevated D-dimer, elevated C reactive protein, elevated troponin, elevated transaminases, elevated IL-6, elevated LDH, presence of pneumonia at the CT or x-ray scan, low platelets levels, use of Venturi’s mask during hospitalization, use of high flow oxygen.

^4^Propensity score analysis included 388 patients with anemia matched for age, gender, presence of comorbidities, oxygen saturation < 92%, elevated procalcitonin, elevated D-dimer, elevated C reactive protein, elevated troponin, elevated transaminases, use of Venturi’s mask during hospitalization, use of high flow oxygen (all parameters with a *p*-value > 0.10) with 388 patients without anemia.

**Figure 2:**
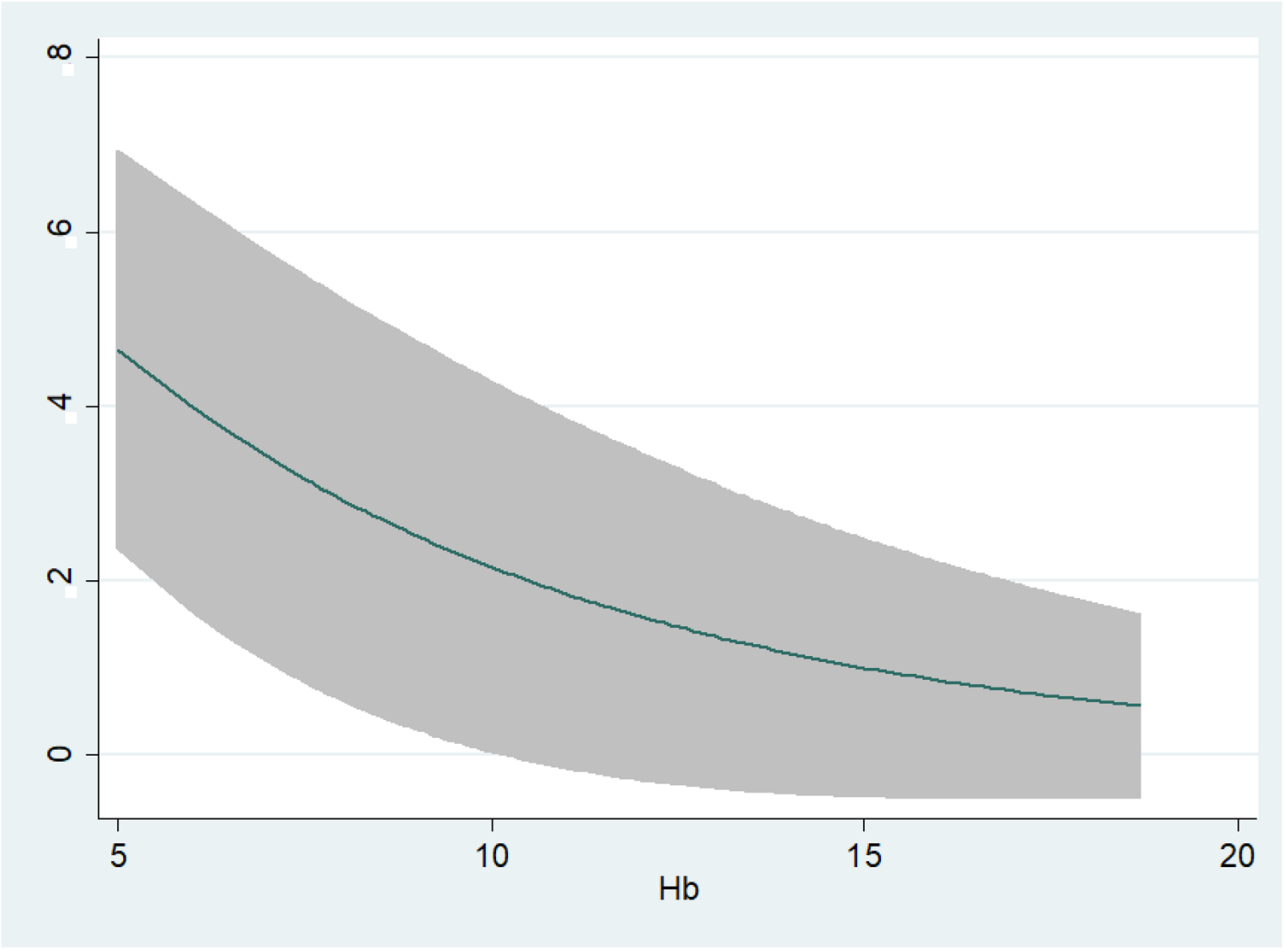
Non-linear association between hemoglobin levels at hospital admission and mortality. X-axis shows hemoglobin levels (mg/dL) at the time of hospital admission. Y-axis shows hazard ratio for in-hospital death.

Figure 2 shows that the association Hb levels at hospital admission and mortality showing a J-shape association. The risk of mortality remained statistically significant higher in patients with a serum Hb levels less than 8 g/dl indicating that more severe forms of anemia are associated with a higher risk of death.

## Discussion

In this study, the overall prevalence of anaemia among patients admitted for COVID-19 was high, affecting about one person over two, and it was independently associated with a higher risk of progression towards severe disease and death even when controlled with a for range of potential confounders. Also, people with anaemia were affected by more underlying chronic conditions and were more likely to present with a baseline biomarker profile suggestive of a more profound activation of the inflammation pathway.

The prevalence of anaemia found in this study is in line with the one found in other cohorts^15,21,22^, and substantially higher than the one found in a meta-analysis including five studies, which found a pooled prevalence of 25.6%^23^. This study confirmed the evidence of anemia as an independent risk factor for severe COVID-19 as observed in other cohorts with smaller populations^24–26^. Interestingly, the prevalence of anemia at admission was similar to the one for community-acquired in both adults^12^ and children^27^ hospitalised for non-COVID community-acquired pneumonia, thus suggesting that this finding may be a characteristic shared also with other lower-respiratory tract infections. Likewise, the association between baseline presence of anemia and the poor clinical outcomes reported here is consistent with other studies involving patients with both COVID-19^28,29^ and non-COVID related pneumonia^12,27^.

In this study, anemic patients were more likely to present with increased levels of C-reactive protein, procalcitonin, lactate dehydrogenase, and ferritin, thus suggesting that the most likely pathophysiological mechanism leading to anemia was inflammation. Also, in our cohort, we found no association between the presence of anemia and higher D-dimer on baseline. This, together with no association between anemia and lower platelet count on admission, point towards ruling out thrombotic microangiopathy from the underlying causes of anaemia in COVID-19.

Furthermore, in our study, anemic patients were more likely to present with lower peripheral oxygen saturation and had a higher prevalence of diabetes mellitus, high blood pressure and chronic kidney disease. In this regard, however—having controlling for both presence of comorbidities, clinical characteristics and baseline inflammation markers—our study provides strong evidence that anemia is a risk factor for disease progression and death in itself, rather than a surrogate of a worse baseline risk. This is supported by the assumption that, in patients affected by pneumonia, low haemoglobin levels both decrease total oxygen content in the blood, at the same time, increase myocardial demand, thus requiring a higher cardiac output to maintain adequate systemic oxygen delivery. Hence, this impaired capability of the cardiorespiratory system to deliver sufficient oxygen to peripheral organs, the need for oxygen supplementation, and the overall risk of death are substantially higher^30^. The role of anemia in increasing the risk of death is highlighted by the observation that, in our study, hemoglobin levels almost linearly associated with an inverse hazard of in-hospital death, where extremely low levels of baseline hemoglobin were associated with approximately a four-fold increased risk of mortality.

This study has several strengths, including a large sample size and a multicentric design. In accordance with the Newcastle–Ottawa Risk of bias assessment for cohort studies^31^, exposed and non-exposed cohorts were drawn from the same population, outcomes were assessed using death certificates and secured medical records, population was truly representative; and correlation to the outcomes were controlled for a wide range of covariates. Also, outcomes were assessed with validated scores. On the other hand, we should acknowledge some limitations. First, being retrospective and observational in nature, its results may be biased by confounding factors that could have affected clinical progression and were not included in the analysis. Second, we did not have information about the presence of any pre-existing anemic condition before the hospitalisation. Third, levels of haemoglobin were assessed only at hospital admission, and we did not report their evolution during hospitalization. Fourth, the study was not designed to investigate the pathological mechanisms leading to anemia in COVID-19. Future studies should focus on prevalence and prognostic role of anemia in other lower respiratory tract infections as well as on providing evidence about the prevalence of anaemia^32^ and its role in guiding therapy for long-term COVID^33^. Finally, we were not able to use Strengths, Weaknesses, Opportunities, and Threats (SWOT) analysis that could better highlights the strengths and limitations of our findings also in terms of economic aspects.

## Conclusions

In conclusion, our study demonstrated that anaemia is highly prevalent in patients hospitalized for COVID-19, and that baseline low haemoglobin levels, even when controlling for an extensive number of potential confounders, are strongly associated with disease progression and mortality. In our view, haemoglobin value, along with other validated risk factors and scores, should be closely monitored in COVID-19 patients requiring hospitalisation to prioritise resources on people at high risk of disease progression and mortality. High-quality evidence about the prevalence and prognostic role of anemia in other lower respiratory tract infections is needed.

## Data Availability

The datasets used and/or analysed during the current study available from the corresponding author on reasonable request.
